# Chemical Composition and Antifungal, Insecticidal and Repellent Activity of Essential Oils From *Origanum compactum* Benth. Used in the Mediterranean Diet

**DOI:** 10.3389/fpls.2022.798259

**Published:** 2022-03-09

**Authors:** Allali Aimad, El Abdali Youness, Rezouki Sanae, Abdelfattah El Moussaoui, Mohammed Bourhia, Ahmad Mohammad Salamatullah, Abdulhakeem Alzahrani, Heba Khalil Alyahya, Nawal A. Albadr, Hiba-Allah Nafidi, Lahcen Ouahmane, Fadli Mohamed

**Affiliations:** ^1^Laboratory of Plant, Animal, and Agro-industry Productions, Faculty of Sciences, University of Ibn Tofail, Kenitra, Morocco; ^2^Laboratory of Biotechnology, Environment, Agri-food, and Health, Faculty of Sciences Dhar El Mahraz, Sidi Mohammed Ben Abdellah University, Fes, Morocco; ^3^Laboratory of Microbial Biotechnology, Agro-Sciences and Environment (BioMAgE), Cadi Ayyad University, Marrakesh, Morocco; ^4^Department of Food Science and Nutrition, College of Food and Agricultural Sciences, King Saud University, Riyadh, Saudi Arabia; ^5^Department of Food Science, Faculty of Agricultural and Food Sciences, Université Laval, Quebec City, QC, Canada

**Keywords:** antifungal, insecticidal, essential oil, drug resistance, bioinsecticide

## Abstract

Essential oils (EO) of *Origanum compactum* Benth. (*O. compactum*) are well known for their biological and pharmacological activities. This study aimed to assess the chemical composition, antifungal, insecticidal and repellent activities of EO of *O. compactum* used in the Mediterranean diet. Phytochemical screening was conducted using gas chromatography-mass spectrometry (GC/MS). Antifungal activity was tested by the disc diffusion method followed by a minimal inhibitory concentration (MIC) assay against *Candida albicans (C. albicans), Aspergillus flavus (A. flavus), Aspergillus niger (A. nige)*, and *Fusarium oxysporum (F. oxysporum)*. Repellent potential and toxicity of EO by contact and inhalation were tested against *Callosobruchus maculatus* (*C. maculatus*). The yield of essential oil obtained by hydrodistillation of *O. compactum* was 4.41 ± 0.35%, mainly composed of Carvacrol (38%) and Thymol (31.46%). Regarding antifungal activity, the results revealed a wide antifungal spectrum of the studied EO against the tested strains, which reached 100% growth inhibition, especially against *A. niger* and *C. albicans* even at the lowest MIC values (3.125 μg/mL). Concerning insecticidal activity, the EO caused total mortality of *C. maculatus* adults at a dose of 20 μL/L air with LC_50_ value of 5.3 μL/L air. A significant reduction in the number of eggs and emergence was proportionally recorded with increasing doses up to 100% at 20 μL/L air. For repellent activity, the studied EO showed a moderate repellent activity with an average percentage of 39.16%. The outcome of this work revealed that *O. Compactum* EO could be a sustainable and environmentally friendly alternative bioinsecticide and bio-fungicide to replace the chemically synthesized forms.

## Introduction

Medicinal and aromatic plants are important sources of EO that find applications in various areas of life. EO is mainly used as a food flavoring but can be successfully used for various non-food applications, as it exhibits many biological properties, including antifungal, antimicrobial, antioxidant, and insecticidal activity (Mssillou et al., 2020; Allali et al., 2021; El Moussaoui et al., 2021).

The compact flowered *Origanum compactum* Benth (*O. compactum)* is one of the most important medicinal plants in terms of ethnobotany in Morocco and southern Spain (Laghmouchi et al., 2018). *Origanum compactum* (Labiatae) is widely used traditionally against several pathologies, with a varied spectrum of use according to region, medication purposes, the parts used, and the mode of preparation (Ennabili et al., 2000; Eddouks et al., 2002). The E O from genus compactum possessed antibacterial (Bouhdid et al., 2008, 2009), antioxidant (Bouhdid et al., 2008), cytotoxic (Babili et al., 2011), antimutagenic (Mezzoug et al., 2007), antifungal (Fadel et al., 2013), and antimalarial properties (Babili et al., 2011).

Chickpea (*Cicer arietinum* L.), is one of the most nutrient-dense seed legumes available for human consumption. It is a good source of protein, vitamins, and minerals (Allali et al., 2020a). Loss of seed yield in pulse crops during storage due to various types of insects, particularly bruchids, is a major issue for farmers (Matos et al., 2020). The chickpea weevil *C. maculatus* (Coleoptera: Bruchidae) is one of the most destructive pest species against chickpea; it can lay eggs in cultivated fields, as well as in storage facilities. The larvae, which feed internally, are difficult to control with chemical insecticides. Fungi in the field and during storage also cause considerable crop losses and deterioration of seed quality (Santos et al., 2016). Contamination by fungi can have serious and dangerous consequences for human health; *A. flavus* produces aflatoxins inducing liver cancer and affecting the growth of young children (Kumar and Kalita, 2017). In addition, invasive candidiasis caused by the candida fungus is frequently associated with high mortality rates, and the emergence of resistant strains (El Moussaoui et al., 2021).

Because of its effectiveness and ease of use, the main approach currently used to control insect pests and fungi in agriculture is the application of synthetic pesticides and fungicides. However, extensive, uncontrolled, and unregulated use of these chemically synthesized products may adversely affect the environment and public health (Amzouar et al., 2016; Kumari and John, 2019; Allali et al., 2020b). On the other hand, synthetic fungicides and disinfectants generally produce chemical residues, which constitute potential environmental pollutants that are difficult to degrade (Gonzalez et al., 2009). It is thus fitting that many recent studies have focused on the search for eco-friendly substances to control pests and microbes without side effects on the environment and public health. In this context, substances of natural origin and particularly EO may represent today an eco-friendly reservoir and more sustainable solution to protect crops.

In this regard, the present work was undertaken to establish whether EO from *O. compactum* (oregano) leaves possess insecticidal and antifungal effects against species of pests and fungi attacking leguminous crops. Thus, in current study, we investigated the chemical composition of EO of *O. compactum* from Taounate, as well as their insecticidal and repellent activities against *Callosobruchus maculatus*, major pests of chickpea grains in Morocco, and the antifungal activity against some pathogenic strains of fungi implicated in the contamination of leguminous and nosocomial infections.

## Materials and Methods

### Plant Material and Extraction of Essential Oils

The leaves of *O. compactum* were used to conduct this work. The whole plant was harvested in June 2020, wherein there was maximum flowering, from the Taunt region (34°30′0″N; 4°33′0″W). The botanical identification of *O. compactum* was carried out by a botanist and given a reference DO12/05005 before being deposited in the herbarium. Next, the leaves were dried in the shade in a dry and ventilated area at a temperature of 25°C for 7 days. The extraction of the EO was performed by hydrodistillation, using a Clevenger-type system according to the manufacturer’s instructions. Briefly, 200 g of *O. compactum* leaves were soaked in 1.25 L of distilled water in a 2-L flask before being boiled for 3 h. The essential oils obtained were dried with anhydrous sodium sulfate and stored in a refrigerator at 4°C until use. The yield was calculated based on the dried weight of the plant using the following formula (1):


(1) $$\text{YHE}=\frac{M\,H\,E}{M\,D}\times 100$$

Where Y_HE_ is the Yield of essential oil (%), M_HE_ is the mass of the EO (g), and M_D_ is the mass of dry plant matter (g).

### Test Insect Collection and Rearing Conditions

The insect *C. maculatus* was collected from a sample of chickpea stored in the city of Fez, Morocco. Bruchids were reared on chickpea seeds (*Cicer aritinum*) packed in glass jars (1 L), covered internally with transparent fabric. The jars were maintained at a temperature of 25 ± 2°C, relative humidity, and a photoperiod of 14 h (light)/10 h (dark) and 65% (± 5%) relative humidity for several successive generations.

### Chromatographic Analysis and Mass Spectrometry

Agilent-Technologies 6,890 N Network GC system with a flame ionization detector and HP-5MS capillary column (30 m × 0.25 mm, film thickness of 0.25 m; Little Falls, CA, United States) was used to analyze the EO. The injector and detector temperatures were set to 250 and 280°C, respectively. The temperature of the column was designed to rise at a rate of 5°C/min from 35 to 250°C, whilst the lower and upper temperatures were kept for 3 and 10 min, respectively. The carrier gas (helium) flow rate was 1.0 mL/min. Using split mode, 1.0 μL of the sample was injected (split ratio, 1:100). The gas chromatograph’s manufacturer offered a built-in data-handling program that was used for all quantifications. The composition was expressed as a proportion of the total peak area. By comparing their GC retention indices, the volatile oil constituents were detected. The mass spectra of each compound were compared to those of the NIST02 GC/MS library data and the Adams library spectra (Adams, 2007).

### Antifungal Activity of Essential Oils

#### Fungal Strains and Culture Conditions

In this study, three filamentous fungi namely *niger, A. flavus*, *F. oxysporum*, as well as one yeast strain *C. albicans* were used for testing reasons. All fungal strains selected are pathogenic and have been associated with drug resistance (Krishnan et al., 2009; Al-Hatmi et al., 2019; El Moussaoui et al., 2021). These strains have been reported as the main producers of mycotoxins and are among the most contaminating microorganisms of dry vegetables and cereals. Spore suspensions were taken from 7-day-old cultures using tubes containing NaCl 0.9%. Afterward, the number of spores in suspension was counted before being diluted to reach an inoculum concentration of around 10^6^ spores/mL (Moussa et al., 2020).

#### Disk Diffusion Method

Assessment of the antifungal activity of *O. compactum* EO was performed by the disc diffusion method (Balouiri et al., 2015). First, Petri plates (90 mm) containing MEA (Malt Extract Agar) medium were inoculated with 0.1 mL of previously prepared microbial culture (10^6^ spores/mL). Thereafter, Wattman paper discs of 6 mm were immediately deposited on the culture media surface after being soaked with 20 μL of EO. Next, the inoculated plates were incubated at 30°C in darkness. Both, inhibition diameter and percent inhibition were determined after 48 h of incubation for *C. albicans* strain and after 7 days of incubation for fungi strains (Zhao et al., 2021).

#### Determination of the Minimal Inhibitory Concentration

In this work, the macro-dilution method was undertaken to evaluate the MICs of *O. Compactum* EO (Moussa et al., 2020). The EOs were immiscible in the culture medium so that their emulsification was conducted using a 0.2% agar solution in order to facilitate germ/compound interaction. To achieve this goal, in sterile hemolysis tubes containing sterile malt extract, broth serial dilutions were made with increasing concentrations up to a final volume of 5 mL in each tube. Consequently, the concentrations of *O. Compactum* EO obtained in the tubes ranged from 100 to 0.09 μg/mL. Next, 100 μL of the media control of each fungal strain was aseptically transferred into each prepared tube except for the media control. Fluconazole FLU (5 mg/mL) was used as a positive control under the same conditions. Finally, the tubes were incubated at 30°C with a rotary shaker for 48 h for yeast and 7 days for fungi. The MIC values of samples correspond to the lowest concentration at which no visible growth was observed in the liquid medium (Bouddine et al., 2012).

### Insecticidal Activity of Essential Oils

#### Toxicity of Essential Oils by Contact Test

Contact toxicity bioassays were performed as described elsewhere (Dutra et al., 2016; Matos et al., 2020) with slight modification. For each EO concentration, 100 g of chickpeas were infested by 5 pairs of insects aged 0–48 h, packed in plastic containers (250 mL) duly closed by a perforated lid, and covered with a thin transparent cloth. Next, EOs were added to the grains using an automatic pipette and then shaken for 2 min. After 48 h of confinement, adult mortality was assessed as reported elsewhere (Dutra et al., 2016). Based on the results obtained in preliminary tests, treatments at different concentrations (1, 5, 10, and 20 μL/100 g) were performed. Parallelly 100 g of chickpeas infested with five pairs of insects without oils were used as control. Dead insects were counted daily until the end of the experiment. Three replicates were performed to measure insecticidal activity, and expressed as a percentage of the average mortality of *C. maculatus* adults before being transformed into corrected mortality by Abbott’s formula (2):


(2) $$\mathrm{Pc}=100\times \frac{P\,0-\mathrm{Pt}}{100-\mathrm{Pt}}$$

Where Pc is the corrected percentage of mortality (%), Po is the observed mortality in the trial, and Pt is the observed mortality in the control.

Eggs laid by females were counted after 12 days from the start of experiment, whilst the emerged individuals were counted after 30 days. The reduction percentage in the number of eggs and adults emerged in each concentration of essential oil was calculated using the following formula (3):


(3) $$\mathrm{PR}=\frac{\mathrm{NC}-\mathrm{NT}}{\text{NC}}\times 100$$

Where PR is the egg-laying or reduction percent of emerged insects (%), NC is the number of eggs or insects hatched in the control and NT is the number of eggs or insects hatched in the treatment.

#### Toxicity of Essential Oils Tested by Inhalation

In the current work, the toxicity of EO was tested by inhalation against *C. maculatus.* To achieve this objective, in 1-L glass jars, small masses of cotton were suspended with a thread attached to the inside of the lid. Doses of 1, 5, 15, and 20 μL/L air of *O. compactum* OE were deposited into the cotton using a micropipette. Afterward, ten *C. maculates* bruchids (male and female) aged between 0 and 48 h were placed in each jar with a perfectly tight seal. For each dose, three replicates were performed. The comparison was made with a control sample (cotton without test solutions).

The Abbott formula (4) was used to calculate the observed mortality rate:


(4) $$\mathrm{Pc}=100\times \frac{P\,0-\mathrm{Pt}}{100-\mathrm{Pt}}$$

Where Pc is the corrected mortality percent (%); Po is the observed mortality in the trial, and Pt is the observed mortality in the control.

### Repellent Activity of Essential Oils

**FIGURE 1:**
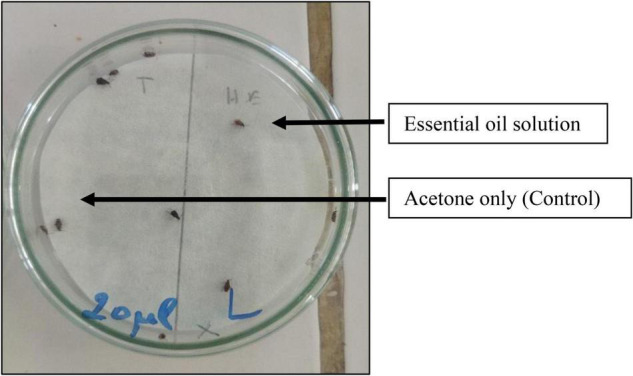
Repellent test of *Origanum compactum* essential oil against *C. maculatus*.

The repellent effect of the essential oil of *O. compactum* against adults of *C. maculatus* was evaluated using the preferential area method on filter paper described by McDonald et al. (1970). Briefly, 9 cm diameter filter paper discs were used for this purpose. These discs were cut into two halves, each with an area of 31.80 cm^2^. For one of the two halves, a volume of 0.5 mL of each EO concentration previously prepared in acetone (1, 5, 10, and 20 μL/mL) was uniformly spread to reach doses of 0.016, 0.079, 0.157, and 0.315 μL/cm^2^ per disk, while the other half received only 0.5 mL of acetone (control). Afterward, the Petri dishes were closed with Parafilm for 30 min. Next, the number of bruchids presented on the half of the disc treated with essential oil was counted against the number of the untreated part. Three replicates for each experiment were done under the same environmental conditions as the insect rearing (Figure 1).

The percentage of repulsion (PR) was calculated according to the following formula (5) (Zandi-Sohani et al., 2013):


(5) $$\text{PR}=\frac{\mathrm{NC}-\mathrm{NT}}{\mathrm{NC}+\mathrm{NT}}\times 100$$

Where PR is the percentage of repulsion (%), NC is the number of insects in the control area, and NT is the number of insects in the treatment area.

The result of the repulsive effects of the essential oil was interpreted according to the classification of McDonald et al., 1970.

### Statistical Analysis

The bioassay experiment followed a randomized design with three replicates for each treatment. Results were expressed as mean ± SE. SPSS for Windows ^®^ statistical software (version 21.0) was used to perform the analyses. Data were assessed by one-way analysis of variance (ANOVA) to determine significant values. Fisher’s minimum significant difference (LSD) test was used as a *post-hoc* test for multiple comparison purposes at α = 0.05. The LC50 and LC95 lethal concentrations with their confidence intervals were determined by the probit method (Finney, 1971).

## Results and Discussion

### Essential Oil Extraction

The EO yield recovered from the Hydrodistillation of leaves of *O. Compactum* was 4.41 + 0.35%. This EO yield was slightly lower than that obtained by Rezouki et al. (2020). Several factors can influence the yield of EO from aromatic and medicinal plants. According to Baranauskienė et al. (2013), the EO production of fresh plants was lower than that of dried plants. This can be explained by increases in the biosynthesis of terpenes and their derivatives after the harvest. This biosynthesis is stimulated by plant water stress and ultimately leads to an increase in the yields of EO. When the plant dies, biosynthetic activity stops, and evaporative losses of EO are no longer compensated, resulting in a drop in distillation yields (Bencheikh et al., 2015). Similarly, Ghasemi et al. (2013) confirmed that drying methods can negatively or positively influence oil content depending on drying time and temperature.

### Gas Chromatography-Mass Spectrometry Analysis

**FIGURE 2:**
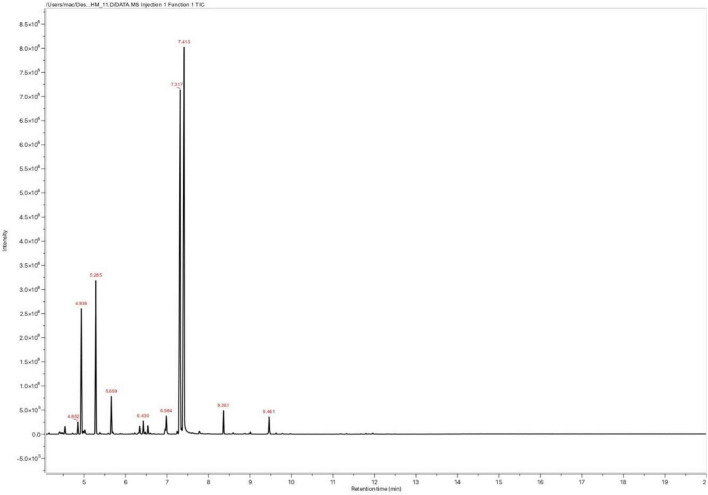
GC-MS chromatographic profile of *O. compactum* EO.

Thirteen compounds were identified in the studied EO by the GC-MS analysis (Table 1 and Figure 2). Carvacrol (about 38.73%), thymol (31.46%), gamma-Terpinene (11.11%), and o-cymene (9.07%) were the major constituents of the EO. The chemical composition of the studied oil was close to that reported by several studies conducted in Morocco. The EO of Moroccan *O. compactum* was characterized by its high content of thymol and carvacrol, which agreed with previous works (Mohammed et al., 2020; Rezouki et al., 2020; Zeroual et al., 2021). The literature has indicated that the yield and chemical composition of EO vary according to the harvesting period, the extraction method, and the drying of the plant, so that our results are in agreement with previous works (Rezouki et al., 2021).

**TABLE 1:** Phytochemical compounds identified in *O. compactum* EO.

Peak	RT	Compound name	RI	Area (%)
1	4.542	Beta-Myrcene	114	0.55
2	4.936	o-Cymene	212	9.07
3	6.341	Borneol L	562	0.60
4	5.285	Gamma-Terpinene	299	11.11
5	5.659	L-Linalool	392	2.41
6	4.852	Alpha-terpinene	191	0.84
7	6.430	4-Terpineol	584	0.90
8	6.538	Alpha terpineol	611	0.58
9	7.317	Thymol	805	31.46
10	9.461	(-) Caryophylleneoxide	1,339	1.21
11	7.413	Carvacrol	829	38.73
12	8.361	Caryophyllene	1,065	1.47
13	6.984	Pulegone	722	1.07
Monoterpenes				97.32
Sesquiterpenes				2.68
Total				100

*RT, Retention time; RI, Retention index.*

### Antifungal Activity

**FIGURE 3:**
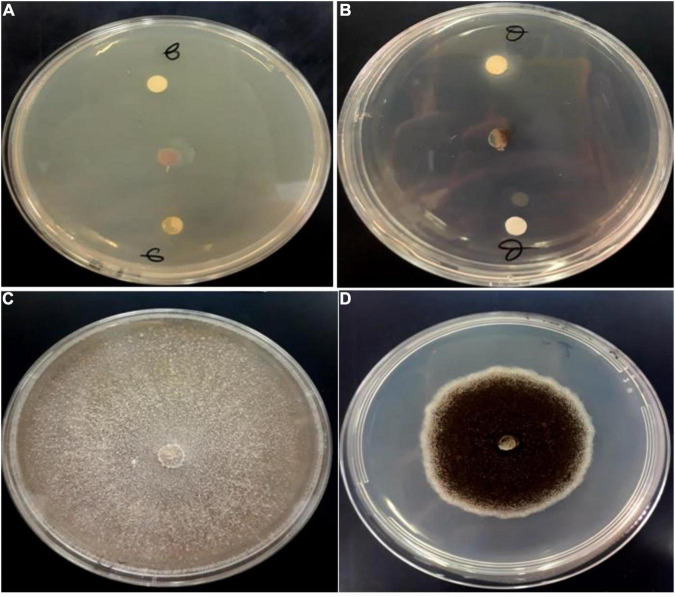
Antifungal activity of *O. Compactum* EO tested by disc diffusion method against *F. oxysporum* **(A)** and *A. niger* **(B)**. **(C,D)** Are untreated fungi for *F. oxysporum* and *A. niger*, respectively (negative controls).

Fungi are frequently implicated in the contamination of leguminous crops during harvest or storage, particularly the genera *Aspergillus* and *Fusarium* (Sud et al., 2005). These mycotoxin-producing filamentous molds along with *C. albicans* are pathogenic and responsible for many fungal infections in hospitalized patients worldwide (El Moussaoui et al., 2021). Figure 3 describes the growth inhibition of the studied fungal strains treated by *Origanum compactum* EOs. The results revealed a high antifungal potential of the studied EOs against the tested strains, marked by a maximum growth inhibition rate (100%).

In addition, the fungicidal effect of *O. compactum* EO was achieved by very low MIC values (Table 2). From this table, it can be seen that *A. niger* and *C. albicans* were the more sensitive to the oil since they were inhibited by the lowest concentration of the EO (3.125 μg/mL). In contrast, higher MICs 6.25 and 12.5 μg/mL were required to inhibit the growth of *A. flavus* and *F. oxysporum*, respectively. On the other hand, EO showed lower MIC values against tested fungal strains when compared to the Fluconazole standard. Our study reported lower MIC values against tested fungal strains when compared to previous work investigating *O. compactum* against *A. niger*, *A. alternata*, *B. cinerea*, *P. digitatum*, *P. italicum*, *V. dahlea*, and *P. expansum*, which were inhibited by MICs ranging from 300 to 450 μg/mL (Mohammed et al., 2020). Another recent study revealed that the MIC values of *Origanum majorana* L.against human pathogenic fungi, including *Candida* species, ranged from 58 to 468 μg/mL (Hajlaoui et al., 2016).

**TABLE 2:** MIC results of *O. compactum* Benth. EO against fungal strains.

Fungal strains	Minimal inhibitory concentration (μg/mL)	
	Essential oil	Fluconazole
*A. niger*	3.125	128
*A. flavus*	6.25	256
*F. oxysporum*	12.5	160
*C. albicans*	3.125	400

The chemical composition of EOs is closely related to their antifungal effect. Potential individual or synergetic effects between the major and minor compounds may occur.

In this respect, some works revealed that *Botrytis cinerea in vitro* mycelial growth and spore germination were strongly inhibited by carvacrol and thymol, the main compound of our Oregano essential oil (Zhao et al., 2021). In addition, other recent research has found that the high content of thymol, carvacrol, γ-terpinene, and *p*-cymene is roughly correlated with *in vitro* and *in vivo* biological activities (Bouyahya et al., 2017). For a better understanding, previous literature has investigated the mechanism of action of EO in fungi. Indeed, the antifungal effect of oregano might be attributed in part to EO terpenes and phenolic compounds involved in cell membrane damage, leakage of cellular materials, inhibition of electron transport, and ATPase in the mitochondria, which ultimately lead to the death of the microorganism (Lagrouh et al., 2017). More comprehensive research revealed that EO of oregano significantly reduced the production of the phospholipase enzyme in *C. albicans* (Brondani et al., 2018).

### Insecticidal Activity of Essential Oils

**FIGURE 4:**
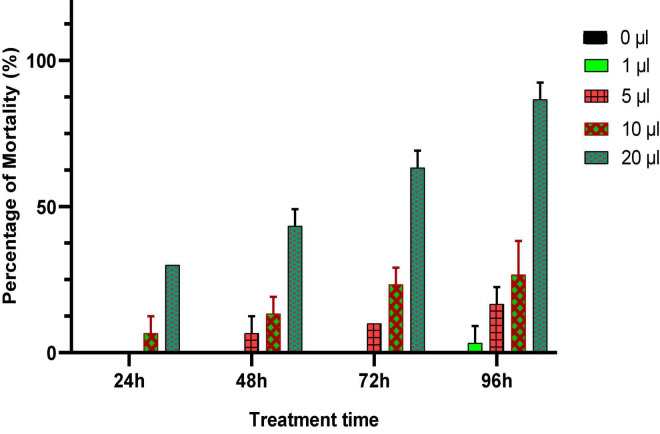
Percentage of mortality (means ± *SD*) *of C. maculatus* adults exposed to an inhalation test of different doses of *O. compactum* EO.

In this experiment, different doses of *Origanum compactum* EO (0; 1; 5; 10; and 20 μL/L air volume) were used to evaluate their toxicity against *C. maculatus* through inhalation. Mortality of adults was noted every 24 h for 4 days, and the results obtained are shown in Figure 4.

According to the results obtained (Figure 4), the oregano EO showed a significant insecticidal effect on the longevity of treated adults. The mortality of *C. maculates* adults increased with increasing doses and duration of exposure to EO. Significant mortality (86.21%) was observed in chickpea bruchid adults treated with a dose of 10 μL/L of oregano EO after 96 h of exposure, which showed the powerful insecticidal effect of the oil.

**FIGURE 5:**
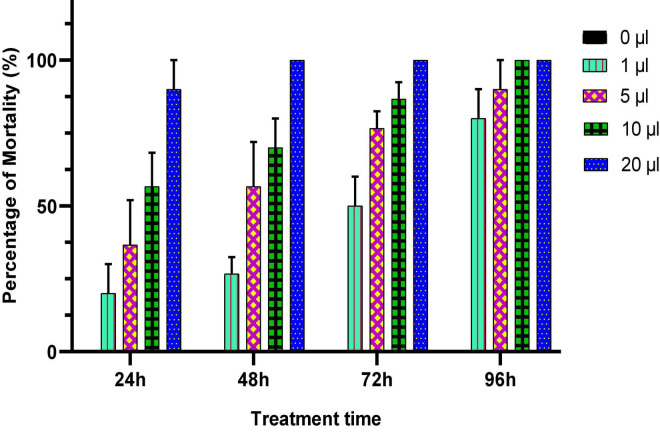
Percentage of mortality (means ± *SD*) of *C. maculatus* adults exposed to a contact test with different doses of *Origanum compactum* EO.

In this test, the EO of *O. compactum* at different doses was applied in direct contact with *C. maculatus* in order to evaluate their toxicity against this pest. The obtained results are listed in Figure 5. Generally, the mortality of *C. maculatus* adults increased when a high dose of the essential oil was applied, and/or when the duration of contact with it approached 96 h. Indeed, at the lowest concentration (1 μL/100 g), the EO of *O. compactum* tested by direct contact caused 80% mortality of *C. maculatus* adults after 96 h of exposure, while at the same concentration tested by inhalation, it caused only 20.69% mortality. At the highest concentration (20 μL/100 g), the tested oils showed significantly higher action when compared to the control and caused 100% mortality with the contact test and 63.33% with the inhalation test after 72 h of exposure. Statistical analysis showed that the LC_50_ and LC_95_ values obtained with the inhalation test (33.61 μL/L air) were higher than those observed with the contact test (5.53 μL/L of air) (Table 3).

**TABLE 3:** LC_50_ and LC_95_ values calculated based on the mortality of *C. maculatus* adults by the inhalation test after 24 h of exposure to *O. compactum* EO.

Bioassays	LC_50_	LC_95_	*X* ^2^
*Inhalation test*	33.61 (24.58;62.83)	211.23 (10.93;1900.8)	1.21
*Contact test*	5.53*	75.67*	17.47

*X^2^, Chi-square.*

*Confidence intervals are too wide, they do not lend themselves to calculation.

**FIGURE 6:**
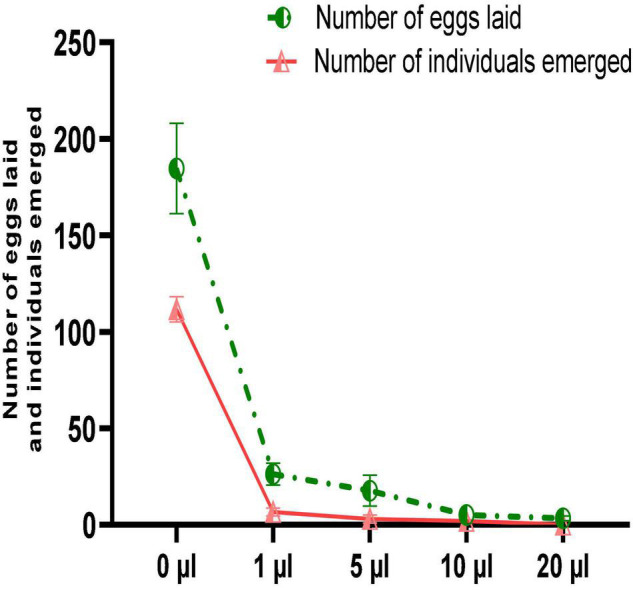
Female fecundity and emergence of new individuals (mean values ± *SD*) after a direct contact test with different doses of EO.

**FIGURE 7:**
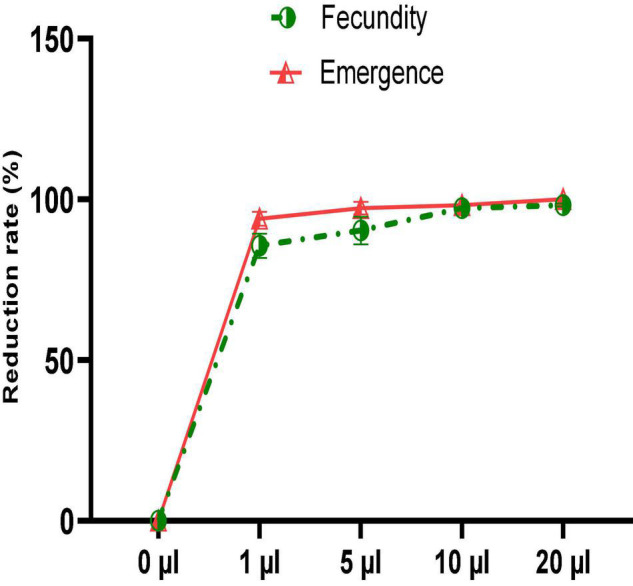
Fecundity and emergence reduction rates after a direct contact test with different doses of EO.

Despite the significant reduction in mortality of *C. maculatus* adults, no oil concentration completely prevented oviposition in females. Figure 6 shows that the number of eggs laid is inversely proportional to the concentration of the EO tested. Thus, at the lowest concentration, the average number of eggs laid per female was 26.33 ± 5.68 representing a respective reduction of 85.7% in egg-laying when compared to the control (Figure 7). At the highest concentration, the average number of eggs laid per female decreased sharply to 3.33 ± 1.15 corresponding to a 98.2% of reduction in oviposition. The number of eggs laid per female of *C. maculatus* in the control jar was 184.67 ± 23.43. For emergence, a significant reduction rate of 100% was observed at the highest dose tested (20 μL/100 g).

## Repellent Activity

The obtained findings figured out that repellent activity was moderate at different doses with a maximum repulsion rate of 56.67 ± 15.26% after 60 min at a dose of 0.315 μL/cm^2^, corresponding to the highest average repulsion rate (39.16%) calculated according to McDonald et al. (1970) (Table 4).

**TABLE 4:** Results of repellent activity of EO from *O. compactum* against *C. maculatus*.

	Doses of EO (μL/cm^2^)				Probability (P)	Average %PR	Class
	0.016	0.079	0.157	0.315			
30 min	13.33 ± 11.55	13.33 ± 11.55	40 ± 20	46.67 ± 11.55	0.03*	28.33	Moderatelyrepellent (II)
60 min	13.33 ± 5.77	33.33 ± 11.55	53.33 ± 11.55	56.67 ± 15.26	0.005**	39.16	Moderatelyrepellent (II)
120 min	0 ± 0	26.67 ± 11.55	46.67 ± 11.55	53.33 ± 11.55	0.0007**	31.67	Moderatelyrepellent (II)

*PR, percentage of repulsion.*

Each number is the mean standard error of three replicates. The symbol * indicates that the difference between the values in the same row are significant, whilst the symbol ** means highly significant (p > 0.05) using the LSD test. Repulsion class: Class 0—0–0.1%; Class I—0.1–20%; Class II—20.1–40%; Class III—40.1–60%; Class IV—60.1–80%; Class V—80.1–100%.

According to the results obtained, *Origanum compactum* EO were effective in the protection of legume seeds. This EO reduced significantly the life span of *C. maculatus* adult bruchids, even at the lowest doses used. This high efficiency resulted in a low value of the LC_50_, 5.53 μL/100 g (contact test) that might be induced by the action of major compounds of these EO (Allali et al., 2021).

Our results showed that the toxicity of EO of *Origanum compactum* increased with increasing doses to reach the maximum at the highest concentrations used. It is therefore appropriate that our results are in agreement with those reported previously (Pavela et al., 2016), which demonstrated that EO of *O. compactum* applied by fumigation on *Tetranychus urticae* adults caused mortality of more than 50%.

Several authors have observed the acaricidal/insecticidal effect of other oregano species. The essential oil of *O. syriacum* is effective by fumigation on *T. cinnabarinus* (Tunc and Şahinkaya, 1998). Aqueous extracts of *O. majorana* were also found to be effective against *T. Urticae* (Pavela et al., 2016). According to Koschier (2008), carvacrol-rich oregano oils show significant activity against several insects, mites, and plant pathogens. For comparison purposes, species among genus *Origanum* have shown significant efficacy against several pests of stored products. For example, *Origanum acutidens* oil rich in carvacrol (87.0%), showed a mortality of 68.3 and 36.7% against two adult insects, *Sitophilus granarius* and *Tribolium confusum*, respectively (Kordali et al., 2008). Moreover, the EO of Oregano has demonstrated strong insecticidal activity against the larvae of *Spodoptera littoralis* with an LC_50_ ≤ 0.05 mL/larva (Pavela, 2015).

Regarding the mode of action of EO on insect pests, a recently published study reported that EO applied by contact on *Sitophilus granarius* insects, pests of cereal seeds, can affect a variety of biological processes in the insects (Renoz et al., 2021). According to these authors, Mentha arvensis oils induced significant physiological changes in exposed insects, particularly on vital functions related to the muscular and neurological systems, cellular respiration, protein synthesis, and detoxification.

## Conclusion

In this present work, chemical composition, antifungal, insecticidal, and repellent actions of EO from *O. compactum* were investigated. In summary, the EO was discovered to be rich in carvacrol and thymol components, which have been remained the primary contributors to pharmacological activities. Consequently, the plant can be a promising source of natural agents with various applications and benefits in health, food, and agriculture. For safety reasons, there is a need to better understand the effect of sublethal dosages of EO on non-target organisms.

## Data Availability Statement

The raw data supporting the conclusions of this article will be made available by the authors, without undue reservation.

## Ethics Statement

The animal study was reviewed and approved by the institutional ethical committee of care and use of the laboratory animals at the Faculty of Sciences Dhar El Mehraz, Sidi Mohamed Ben Abdallah Fes University, Morocco, reviewed and approved the present study # 04/2019/LBEAS.

## Author Contributions

AAi and EY: writing—original draft preparation. RS and AE: formal analysis. MB, AMS, AAl, HKA, H-AN, and NA: writing—reviewing and editing. LO and FM: supervision and data validation. All authors contributed to the article and approved the submitted version.

## Conflict of Interest

The authors declare that the research was conducted in the absence of any commercial or financial relationships that could be construed as a potential conflict of interest.

## Publisher’s Note

All claims expressed in this article are solely those of the authors and do not necessarily represent those of their affiliated organizations, or those of the publisher, the editors and the reviewers. Any product that may be evaluated in this article, or claim that may be made by its manufacturer, is not guaranteed or endorsed by the publisher.
